# How to Study the City on Instagram

**DOI:** 10.1371/journal.pone.0158161

**Published:** 2016-06-23

**Authors:** John D. Boy, Justus Uitermark

**Affiliations:** 1 Sociology Department, University of Amsterdam, Amsterdam, The Netherlands; 2 Sociology Department, Erasmus University, Rotterdam, The Netherlands; University of Warwick, UNITED KINGDOM

## Abstract

We introduce Instagram as a data source for use by scholars in urban studies and neighboring disciplines and propose ways to operationalize key concepts in the study of cities. These data can help shed light on segregation, the formation of subcultures, strategies of distinction, and status hierarchies in the city. Drawing on two datasets of geotagged Instagram posts from Amsterdam and Copenhagen collected over a twelve-week period, we present a proof of concept for how to explore and visualize sociospatial patterns and divisions in these two cities. We take advantage of both the social and the geographic aspects of the data, using network analysis to identify distinct groups of users and metrics of unevenness and diversity to identify socio-spatial divisions. We also discuss some of the limitations of these data and methods and suggest ways in which they can complement established quantitative and qualitative approaches in urban scholarship.

## Introduction

Since its launch in 2010, Instagram has quickly become one of the most widely used social networking platforms in the world [1]. In early 2015, it was reported that over 200 million users around the world use the service to share 70 million pictures per day [2]. As a visual-locative social medium, Instagram can be regarded as a participatory sensing system [3,4]. Its users produce data as they navigate their everyday lives, smartphones in hand. These data are centralized in the Instagram platform. As such, it lends itself as an unparalleled data source for social researchers. In this paper, we introduce methods that can be used by scholars to make sense of contemporary urban dynamics. In particular, our interest is to explore and visualize sociospatial patterns and divisions within the city. We apply these methods to two cities, Amsterdam and Copenhagen. This application is a proof of concept to demonstrate the possibilities—as well as the limitations—of these data and methods to inform the work of urban scholars.

## Related Work

Our approach for studying social divisions draws on two literatures: social science literature on cities, and computer science literature on social media. Social scientists have long been interested in social divisions within the city. Building on the foundational work on urban social ecology by the Chicago School [5], they have mapped the uneven distribution of population groups and identified determinants and outcomes of economic and ethnic segregation [6–8]. The vast majority of studies have researched residential segregation through official registry data. While such studies provide a range of insights, it is widely acknowledged that these studies do not cover some essential dimensions of differentiation within the city, as they account more for where people reside than for how they move through urban space in their daily lives. The urban landscape is marked by numerous processes of enclave formation that do not revolve around places of residence, i.e. neighborhoods, but around places of work, leisure, or retail [9–13]. Researchers have relied on ethnography or survey research to grasp these subtle and dynamic patterns of segregation [14,15]. While these methods provide distinct advantages, they are arguably less suited to capture relations and flows outside the bounds of the research site [16–18].

User-generated data from location-based social networking platforms hold the promise of filling in the gaps in the picture presented by current research on urban dynamics [19]. As the product of a participatory sensing system, these data add to the methodological toolkit of scholars of the city. These data can be used to identify groups on the basis of observed behavior rather than using a predefined classificatory scheme, allowing for a more fine-grained and up-to-date breakdown of urban populations into subgroups.

However, researchers seeking to put these opportunities to use should be aware that social media data do not simply reflect the activities of urban dwellers. This is especially true for the Instagram data we utilize in this paper. Instagram users selectively represent their lifeworlds by showcasing images they feel are suited for circulation. This also means that they represent the city and their place within it in a curated manner. Users typically do not report on their visits to the supermarket or their commute to work. Instead they share images as part of strategies of distinction: they picture themselves with friends, in nice outfits, in places that are special to them [20]. In a word, they use Instagram to mark their place in the social structure and within the city. By associating with each other (by following, liking, or commenting) and tagging the same places, users form communities at the interface of online and offline spaces. By mapping these processes of association and place demarcation, we can investigate how communities emerge at this interface and create sociocultural domains. Such processes could before only be grasped through surveys or ethnographies of concrete settings, but now we can use social media data to investigate on a large scale and in detail how city dwellers associate with one another and form communities.

These data can also give insight into the places or sets of places in which different groups in the city spend their waking hours and into the role these places perform in the formation of groups and subcultures. In this context, Lofland understands a city’s public realm to be made up of places in which city dwellers encounter strangers [21]. These places are quintessential sites of urban life because they require people to interact with others with which they have no intimate bonds [22]. As such, they also serve as sites to cultivate cosmopolitan habits [23]. Urban dwellers can also transform nominally public places into a group-specific domain. Such parochial places serve to solidify group identities and reaffirm boundaries [21]. Thus, identifying which places in a city are cosmopolitan and which are parochial is important for an understanding of patterns of encounter and enclavement in the city. In addition, divisions between groups can also occur in time rather than in space, for instance when places and areas become exclusive sites in a city’s nightlife [24–27].

While several scholars working at the juncture between geography and computer science have begun using social media data [28–31], within the last half decade computer scientists have conducted most of the work taking advantage of location-based social networks [32]. Before the recent rise of Instagram, the focus was on geotagged tweets and data sourced from Foursquare, the check-in service. Cranshaw et al.’s Livehoods Project presents a method to study urban dynamics and structure through social media data using machine-learning techniques [33]. Their methodology aggregates individual data points (Foursquare checkins) using spatial clustering techniques to identify areas that emerge from the actions of city dwellers. Frias-Martinez et al. use geotagged tweets to study land use and sites of interest in New York City. On the basis of spatio-temporal patterns in the data, they distinguish areas in the city used primarily for leisure, business, or residential purposes [34]. Silva et al. use Foursquare data to create heatmaps to visualize urban dynamics on the basis of individual trajectories. The resulting visualization shows the overall likelihood of a city’s inhabitants of transitioning between different types of spaces, such as public transit hubs and places of work [35,36].

More recently Silva et al. have moved from using Foursquare data to using Instagram data as the basis for a “participatory sensing system.” (In [37] Silva et al. compare the two data sources.) In using Instagram data to study urban dynamics, they find spatio-temporal patterns to be correlated with routine activities of city dwellers. As such, it can serve to identify places or sets of places of cultural activity [38]. A series of interdisciplinary collaborations between computer scientists and art historians have also sought to make sense of Instagram in the context of the city. By analyzing large datasets of geotagged posts collected in cities throughout the world, these contributions seek to visualize differences in rhythms and content between cities [39–41]. Other contributions by researchers around Lev Manovich include http://selfiecity.net and http://on-broadway.nyc.

In sum, while scholars have undertaken promising forays, research so far has been limited. Especially when compared to Twitter, research on Instagram is in its infancy. There are important theoretical reasons for filling this lacuna, as Instagram data enable researchers to shed new light on processes that have long occupied scholars of cities, including the formation of subcultures, segregation, and the cosmopolitan or parochial nature of places within the city. Our contribution is to develop a number of methods to illuminate these processes and to provide a proof of concept for how these methods can be put to work.

## Data

We utilize both network and spatial data sourced from Instagram. Network data allow us to identify groups, while spatial data allows us to map the places that Instagram users picture. Taken together, we can use this data to identify socio-spatial divisions by investigating the presence or absence of social groups in places throughout the city.

We collect both kinds of data from Instagram using the platform’s application programming interface (API). For this purpose we built and used kijkeens [42], a tool that polls the Instagram API’s location endpoint at regular intervals to gather all geotagged posts from an urban area, stores post metadata in a database, and, after a specified delay, gathers network data (“likes” and comments) for each of the posts. We created two dataset of posts published in Amsterdam and Copenhagen over a twelve-week period. In Amsterdam we collected 953,403 posts between 19 April and 12 July 2015, while in Copenhagen we collected 890,621 posts between 25 May and 17 August 2015. Because we are interested in everyday patterns of urban dwelling, we only considered posts by users who had posted in the city over a time spanning four weeks or longer to eliminate likely tourists. This cut the number of posts down to 442,246 and 507,445 posts, respectively.

We stored a variety of metadata about each of these posts. Most importantly for our purposes here are the data about social activity (“likes” and comments) and tagged locations. We stored the social activity on each post about 24 hours after the initial publication of the post. Since most of the activity on a post occurs within the first few hours of its life, this accounts for the bulk of the interactions garnered by the geotagged posts in our dataset. In Amsterdam, there were over 16 million interactions, of which 1.1 million were between local users in our dataset. In Copenhagen, the number of interactions was over 21 million, of which 1.8 million were between users in our dataset. It bears keeping in mind that the API returns a maximum of 140 likes per post, so we cannot capture all interactions for very popular users whose number of likes regularly exceeds this number. As a result of this restriction of the data, our analysis may underestimate the centrality of certain users in the overall network.

## Methods

Our analysis combines several methods. We use network analysis to identify groups and metrics of unevenness and diversity to identify socio-spatial divisions.

### Identifying Groups Using Network Analysis

How can we identify groups of city dwellers? Where previous scholars had to collect data for their analyses through painstaking community studies [43,44], we are able to use the network data captured on Instagram. Instagram users give recognition to others on the platform by liking and commenting on their posts. For the purposes of our network analysis, we understand reciprocated recognition (mutual liking and/or commenting) to constitute a social tie between two users. Research on social media use suggests that this provides a surer indicator of a social tie between users than mere followership [45]. We construct an undirected, unweighted network graph on the basis of our interaction data. Table 1 provides some metrics on the two city networks.

**Table 1:** Networks of Mutual Ties in Amsterdam and Copenhagen.

City	Total Nodes	Nodes With Reciprocated Ties	Nodes in Largest Component	Edges	Median Number of Ties	Modularity	Clusters
Amsterdam	30,964	20,135	18,916	56,126	3	0.635	12
Copenhagen	41,232	32,339	31,227	98,978	4	0.618	16

We identify subgroups among Instagram users by applying a technique called community detection. The method we use, called the Louvain method of modularity optimization [46], progressively groups connected nodes in a network together until it reaches an optimal level of clustering. We use the igraph package [47] and the implementation of the Louvain algorithm by Traag [48]. We perform community detection on the largest connected component of each graph, which accounts for most nodes with reciprocated ties in both networks (93.9 percent for Amsterdam and 96.6 percent for Copenhagen). We consider only clusters of at least five hundred users. We chose this cutoff to keep the number of clusters manageable after determining that the clusters above this cutoff contain most nodes. Those interested in studying specific subcultures may want to include even some of the smaller, more marginal clusters, but that is not necessary for our purposes.

Next, we characterize these groups and find what, aside from the overall network structure, makes them distinct. We tried out a variety of methods. At first, we sought to characterize clusters in an automated manner by using user profile data. Instagram users have the possibility of filling in a 150-character “biography” field. Our attempts to use text analysis techniques such as tf-idf [49] to characterize clusters on the basis of this textual data failed to yield reliable or valid results. Instead, we opted to rely on a combination of network analysis and manual classification to characterize groups. Despite some shortcomings, this seems the most suitable approach given the data we have.

In a first step, we analyze the structure of subgraphs. We are particularly interested in the density of ties and in whether certain nodes stand out as hubs. If we find that subgraphs are tightly knit and organized around hubs, then we have a rationale for characterizing groups in terms of their most central users, which we can regard as group focal points [50]. To determine tie density of subgroups, we calculate the local average clustering coefficient and compare it to the clustering coefficient of a random Erdős–Rényi graph with an equal number of nodes and edges [51]. We report a logged ratio of clustering coefficients ($log\frac{C_{subgraph}}{C_{random}}$) to compare observed tie density to that of a random graph. If the clustering coefficient is significantly higher than in the random graph, we can conclude that tie density is high. To determine the extent to which subgraphs are organized around hubs, we inspect the centrality distribution of each subgraph and report goodness-of-fit measures (Kolmogorov-Smirnov distance) for three heavy-tailed distributions: power law, lognormal, and stretched exponential distributions [52]. We use Page Rank as a measure of network centrality [53].

As we will see, the subgraphs have a skewed, heavy-tailed centrality distribution (indicative of hubs) and high tie density (as compared to random graphs). We exploit these network features and characterize groups according to their focal points. We did so manually. The authors each looked at the data to inductively arrive at a characterization for each cluster and then compared results to fine-tune the results of this inductive process. Future research may want to develop a coding scheme for these purposes, but that is not something we could draw on here. In characterizing these central accounts, we first consider the user profile, and then we analyze the content of their pictures and the tagged locations. Often users list their profession or affiliation in their biography which we then only have to verify by studying their images, but other times we have to determine their social and cultural background through close study of the content of their images. Our analysis focused on the ten most central accounts. While a more exhaustive manual analysis undoubtedly would have revealed further nuances, we found that examining the ten most central accounts provided us with a good impression of the cluster in the sense that examining additional accounts did not lead us to fundamentally change our characterization.

### Mapping Social Divisions and Interactions

How can we measure the level and nature of segregation and interaction between groups? Sociological studies of urban segregation have employed a number of different metrics. The index of dissimilarity (DIS) was long considered the gold standard of residential segregation measures [54], and it is particularly suited to capture the evenness of populations in an urban area [55]. The segregation of a minority group M across k different areal units i relative to a majority population W is measured as follows:

$$\text{DIS}=\frac{1}{2}\overset{\text{k}}{\underset{\text{i}=1}{\sum }}\left|\frac{{\text{m}}_{\text{i}}}{\text{M}}-\frac{{\text{w}}_{\text{i}}}{\text{W}}\right|$$

Here m_i_ and w_i_ refer to the subpopulations of the minority and minority populations found in each areal unit. This index has a variety of characteristics to recommend it. Above all, it is easy to interpret. The value of DIS corresponds to the proportion of the minority group that would have to relocate to achieve a fully even distribution. We use the dissimilarity index to measure group segregation, since we are interested in how evenly groups are present in places around the city. In calculating this measure of evenness, we take each cluster as a “minority group” and compare it to the other clusters combined forming the majority group.

We are also interested in exchanges between groups. Two groups can be related to one another by being in frequent interaction with one another, for instance by liking and commenting on each other’s posts. The strength of this relation is indicated by the weight of the edge connecting the two groups in the cluster graph. The edge weight is calculated by summing up reciprocated ties between members of both groups. We normalize edge weights by dividing an edge’s weight by the combined number of nodes in the two clusters it connects.

### Locating Cosmopolitan and Parochial Places

Which places facilitate encounters between members of different groups, and which are exclusive to members of the same group? We rank places from most parochial to most cosmopolitan by employing a diversity measure known as the divergence index. It compares the expected distribution of groups in a place given what we know about the overall distribution of these groups in the city as a whole to the observed distribution within that place. Instagram users can tag their posts with predefined locations, but they can also define their own place names (or at least they could during our period of data collection). We manually verified and, where necessary, merged place names. We only consider places tagged in at least 25 posts by at least 15 different users. If each group was represented in the proportion in which it is present in the city, we would have a situation of full diversity. The value of the measure would be 0. The further the expected and observed distributions diverge in a particular place, the less diverse that place can be said to be. The divergence index (DIV) is used in both the social sciences and the life sciences to measure population diversity [56,57]. The divergence index in areal unit i is defined as follows:

$${\text{DIV}}_{\text{i}}=\overset{\text{s}}{\underset{\text{m}=1}{\sum }}\pi_{{\text{i}}_{\text{m}}}\text{log}\frac{\pi_{{\text{i}}_{\text{m}}}}{\pi_{\text{m}}}$$

In this equation, π_m_ corresponds to the overall relative occurrence of cluster m, and $\pi_{i_{m}}$ refers to the relative occurrence of cluster m in areal unit i. For ease of interpretability, we report standardized divergence indices, which we calculate by dividing DIV_i_ by max(DIV) [56]. A value of 1 thus indicates maximum divergence (i.e., lowest diversity).

Unlike other diversity measures based on concepts from information theory, DIV is not impacted by the number of subgroups being considered [56]. This is important because network structures differ between cities, so community detection can yield a different numbers of subgroups. If we want diversity measures to be comparable, they must not be influenced by how many communities there are.

## Results

### Groups

Community detection allowed us to identify twelve clusters of 500 or more users in Amsterdam (Tables 2 and 3) and sixteen clusters above the cutoff in Copenhagen (Tables 4 and 5). The users in these clusters account for more than two thirds of all users with reciprocated ties in the case of Amsterdam and more than three quarters of users in the case of Copenhagen.

**Table 2:** Clusters detected in Amsterdam.

Cluster	Posts	Users	Posts per User (Mean)	Followers (Median)	Private Accounts (%)
AMS1	2,287	30,565	13.4	462	15.6
AMS2	2,115	49,424	23.4	525	4.2
AMS3	1,908	49,858	26.1	319	3.0
AMS4	1,846	34,888	18.9	456	5.0
AMS5	1,245	16,635	13.4	365	18.4
AMS6	768	10,557	13.7	366	19.2
AMS7	872	12,151	13.9	431	12.8
AMS8	694	8,034	11.6	309	8.8
AMS9	693	11,831	17.1	295	3.7
AMS10	604	10,061	16.7	259	5.8
AMS11	595	9,753	16.4	284	6.1
AMS12	523	8,323	15.9	251	6.3

**Table 3:** Additional data on Amsterdam clusters.

Cluster	Label	DIS	Power Law Fit	Lognormal Fit	Stretched Exponential Fit	Clustering Ratio
AMS1	Urban Street Culture	0.464	0.069	0.021	0.020	1.69
AMS2	Lifestyle Vanguard	0.332	0.041	0.029	0.038	1.74
AMS3	City Imagers	0.368	0.033	0.012	0.025	1.77
AMS4	Cultural Entrepreneurs	0.297	0.057	0.020	0.026	1.68
AMS5	High School Students	0.434	0.075	0.023	0.023	1.68
AMS6	unclear	0.409	0.050	0.036	0.048	1.57
AMS7	Party Buffs	0.465	0.053	0.018	0.026	1.55
AMS8	Teenage Bon Vivants	0.306	0.054	0.035	0.048	1.52
AMS9	Visual Professionals	0.336	0.062	0.032	0.038	1.64
AMS10	Cultural Explorers	0.405	0.034	0.035	0.047	1.47
AMS11	Health & Lifestyle Devotees	0.349	0.051	0.052	0.070	1.44
AMS12	Coffee Aficionados	0.356	0.058	0.050	0.070	1.44

**Table 4:** Clusters detected in Copenhagen.

Cluster	Users	Posts	Posts per User (Mean)	Followers (Median)	Private Accounts (%)
CPH1	4,445	73,803	16.6	303	3.7
CPH2	3,605	32,514	9.0	325	7.2
CPH3	2,733	23,727	8.7	410.5	10.4
CPH4	2,766	61,462	22.2	321.5	2.5
CPH5	2,421	41,982	17.3	188	3.6
CPH6	2,339	23,662	10.1	246.5	6.4
CPH7	2,268	35,012	15.4	221	4.4
CPH8	818	12,053	14.7	251	8.6
CPH9	798	5,778	7.2	315	6.7
CPH10	774	9,197	11.9	197	4.8
CPH11	727	9,829	13.5	195	4.6
CPH12	654	8,483	13.0	221	7.0
CPH13	634	7,187	11.3	262	5.9
CPH14	622	7,030	11.3	183.5	4.8
CPH15	552	6,370	11.5	189.5	5.0
CPH16	513	7,135	13.9	171	4.3

**Table 5:** Additional data on Copenhagen clusters.

Cluster	Label	DIS	Power Law Fit	Lognormal Fit	Stretched Exponential Fit	Clustering Ratio
CPH1	Designers	0.335	0.051	0.014	0.019	1.942
CPH2	College Students	0.300	0.050	0.033	0.046	2.066
CPH3	Gymnasium Students	0.379	0.052	0.031	0.043	1.998
CPH4	Design & Family	0.315	0.035	0.016	0.028	1.852
CPH5	Photographers	0.305	0.027	0.017	0.034	1.844
CPH6	College Students	0.267	0.046	0.023	0.032	2.056
CPH7	Fitness & Nutrition	0.366	0.034	0.013	0.033	1.925
CPH8	Teenagers	0.504	0.066	0.042	0.050	1.793
CPH9	Gymnasium Students	0.364	0.056	0.024	0.027	1.673
CPH10	unclear	0.314	0.047	0.042	0.047	1.735
CPH11	Political Activists	0.326	0.040	0.022	0.038	1.692
CPH12	Urban Street Culture	0.359	0.056	0.052	0.064	1.598
CPH13	High-Brow Culture	0.337	0.053	0.036	0.048	1.622
CPH14	unclear	0.376	0.072	0.068	0.085	1.645
CPH15	Fashion & Marketing	0.353	0.075	0.038	0.051	1.485
CPH16	Yoga & Family	0.391	0.043	0.038	0.053	1.464

All cluster subgraphs have clustering coefficients that are significantly higher than in a corresponding randomly generated graph. For all clusters, the difference exceeds an order of magnitude. This points to the high density of ties within the clusters. Furthermore, their centrality distributions hew closely to a heavy-tailed distribution (see also S1 Fig), which speaks to the existence of hubs within each cluster. This provides us with a rationale to characterize groups in terms of their most central users.

In the case of Amsterdam, we are able to characterize eleven out of twelve clusters using manual classification of central users. The groupness of the clusters generally is rooted in shared professions, interests, lifestyles, and hangouts. Some clusters, such as AMS5 (consisting mostly of high school students), have no strong tie to particular places and no common ways of identifying. They are, however, at a similar stage in their lives. We might say that the young people in this cluster have not (yet) developed a distinctive style or autonomous identity. Finally, AMS6 –a cluster with an unusually high proportion of private accounts—has no basis for groupness that we can discern.

In Copenhagen, a greater number of clusters is characterized by shared stage of life rather than shared professions or hangouts. Part of the reason is that more teenagers are visible on Instagram in Copenhagen than in Amsterdam, indicating either a higher level of adoption of the social network among Danish youth or a lower aversion to setting accounts to public and using geotags. Nonetheless, several clusters are clearly defined by shared characteristics, interests or professions.

Comparing Amsterdam to Copenhagen, we can see some commonalities and differences. Both cities contain a sizeable cluster of image makers whose main occupation on the platform, whether vocationally or avocationally, is to picture the city in which they are based (AMS3 and CPH5). Places tagged by users in these clusters include well-known parks, buildings and structures. In both cities, clusters vary in size, in degrees of activity, and in popularity. The most active cluster in Amsterdam (AMS3) has an average of 26.1 geotagged posts per user during the twelve-week period under investigation and the least active (AMS8) has 11.6 posts per user. In Copenhagen, the most active cluster (CPH4) has an average of 22.2 geotagged posts per user, while the least active (CPH9) has only 7.2. Finally, the number of followers also varies between clusters. On average, follower numbers in Amsterdam are higher than in Copenhagen, and the spread of follower numbers is also greater. The Lifestyle Vanguard in Amsterdam (AMS2) has the highest average number of followers, and in contrast, the most popular Copenhagen cluster (CPH3) consists of high school students.

### Social Divisions and Interactions

**Fig 1:**
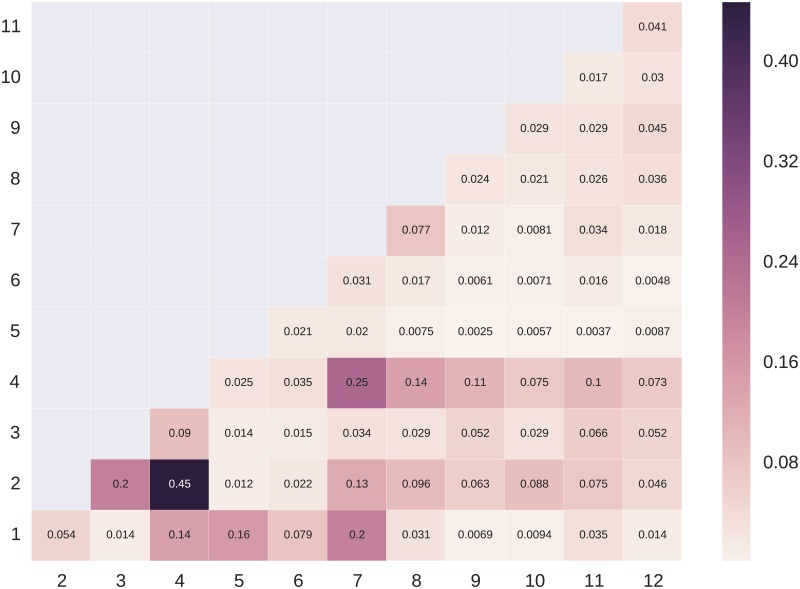
Levels of interaction between clusters in Amsterdam. Cells show the average number of mutual ties per user between members of two clusters. Darker shaded cells indicate a greater number of mutual ties. Blank cells indicate an absence of interaction.

**Fig 2:**
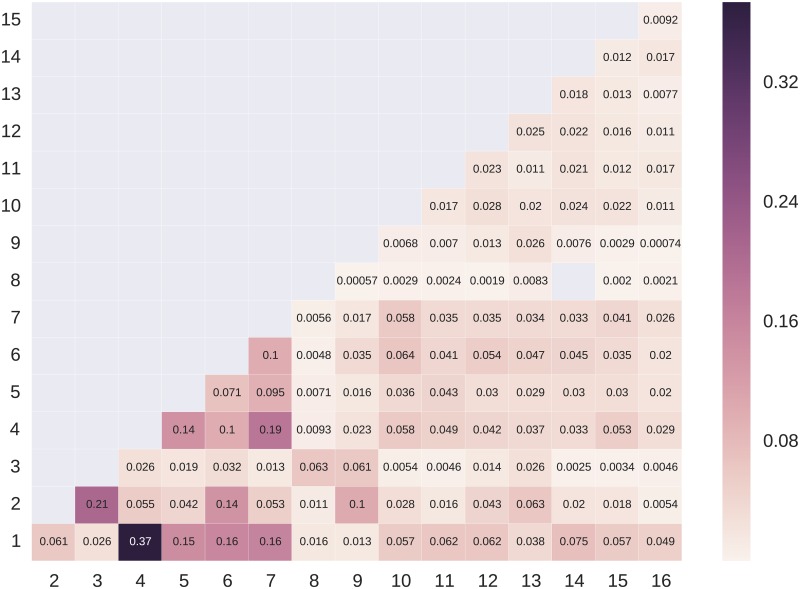
Levels of interaction between clusters in Copenhagen. Cells show the average number of mutual ties per user between members of two clusters. Darker shaded cells indicate a greater number of mutual ties. Blank cells indicate an absence of interaction.

As indicated by the dissimilarity index (DIS) reported in Tables 2b and 3b, the presence of clusters in places around the city is uneven. To achieve an even groupwise distribution of posts, on average about one in three posts would have to be posted from elsewhere. The uneven presence of clusters throughout the city does not mean that they are completely isolated, however. Figs 1 and 2 show levels of interaction between clusters in the two cities.

In Amsterdam, clusters AMS2 and AMS4 have 0.45 mutual ties per user, the strongest tie between two clusters. Given the similarity in lifestyle orientations between the two clusters—one consisting of the Lifestyle Vanguard, the other consisting of Cultural Entrepreneurs—this link is not too surprising. The next strongest tie is between clusters AMS4 and AMS7, the Cultural Entrepreneurs and the Party Buffs, another pair of clusters whose lifestyles, while not overlapping, appear to have an affinity. Clusters AMS1 and AMS3 have strong ties to AMS7 and AMS2, respectively. These five interconnected clusters are among the most popular, and they are each defined by shared lifestyles and professions rather than a common stage of life.

In Copenhagen, clusters CPH1 and CPH4 have a strongest tie, with 0.37 mutual relations per user. Both of these clusters are firmly grounded in design professions and affinities, though CPH4 has a higher degree of young parents whose family life appears on Instagram alongside their interest in design. Clusters CPH2 and CPH3, large clusters consisting of men and women in their teens and twenties attending secondary and postsecondary education institutions, also have a strong tie.

### Cosmopolitan and Parochial Places

**Fig 3:**
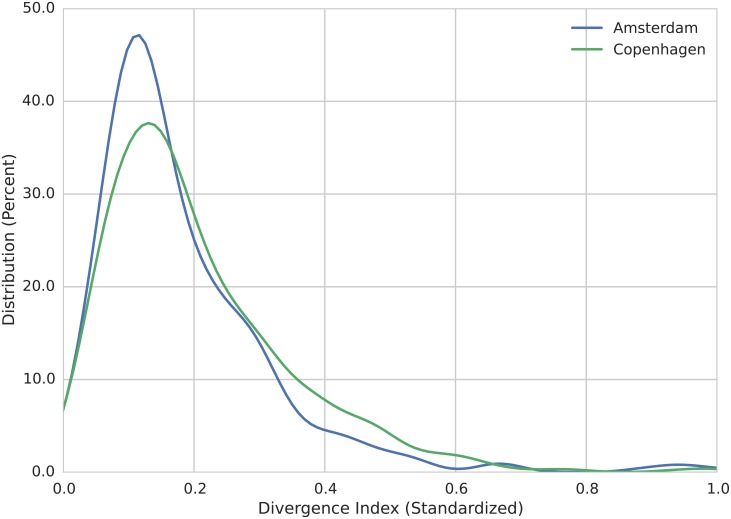
Distribution of divergence indices in Amsterdam and Copenhagen. The divergence index is inversely proportional to the diversity of a place. We calculate the index for each place in the two cities in our study. The graph shows the distribution of standardized scores.

Users tagged 367 places in Amsterdam and 680 places in Copenhagen. The following visualizations show the places in order of diversity, focusing only on the thirty most tagged locations for ease of presentation (Fig 3 shows the distribution of the divergence indices for all tagged places in both cities). This way of presenting our data has the benefit of making patterns of group co-occurrence in places easily apparent. The most diverse places are tagged by members of all clusters, suggesting they are places of encounter between a wide array of different groups (i.e., cosmopolitan places). The least diverse are the exclusive domain of just one or two clusters (i.e., parochial places). Those with middling levels of diversity are overwhelmingly tagged by members of the same three to four groups. They are neither exclusive parochial domains, nor are they places of broad encounter.

**Fig 4:**
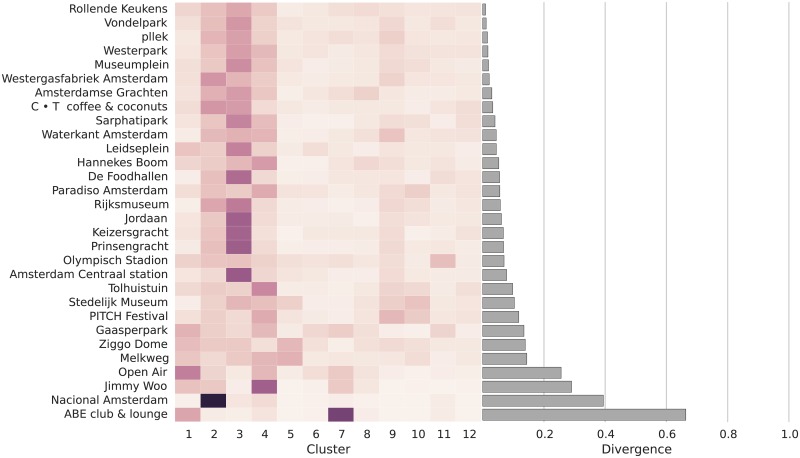
Visualization of place diversity in Amsterdam. The horizontal bar graph on the right shows the value of the divergence measure (DIV), with higher values indicating lower diversity. The heatmap in the middle indicates the relative presence or different clusters. Darker shaded cells shaded indicate greater presence of a cluster; white cells indicate complete absence.

The Rollende Keukens (“rolling kitchens”) open air food cart festival in Amsterdam has near-proportional representation from all twelve clusters of Instagram users (see Fig 4). Like some other “places” we find in our data, the festival is actually a temporary happening, which in this case took place in the Westerpark, a public park in the west of Amsterdam. The location of the event might explain its broad appeal, as the list of the most cosmopolitan places includes the Vondelpark, Westerpark, Westergasfabriek (a cultural center located in the Westerpark), and the Museum Square—all public places. The parks and squares which Amsterdam Instagram users fondly picture and associate themselves with frequently become sites of encounter between different groups.

These public places are followed by popular cafes and other hangouts, an independent concert venue, and two of the city’s famous art museums. Several of these places have the strong presence of AMS3, the cluster of City Imagers who picture famous sights. Clusters AMS2 and AMS4 are also a constant presence, while clusters AMS5 and AMS6 are absent from several of them. As we move closer to the parochial end of the spectrum, there is a marked increase in music festivals, clubs, and large concert venues. Several of these places have a strong presence of members of clusters AMS1, AMS4, and AMS7. The most parochial places, which include restaurants, clubs, and a gym frequented by Health and Lifestyle Devotees (AMS11), are the preferred hangout of members of just a single cluster. The type of places featured among the most parochial suggest that the city is particularly segregated at nighttime, since they include several nightlife locations. Some clusters are completely absent from the most parochial places, including the Visual Professionals (AMS9), Cultural Explorers (AMS10), and the Coffee Aficionados (AMS12).

**Fig 5:**
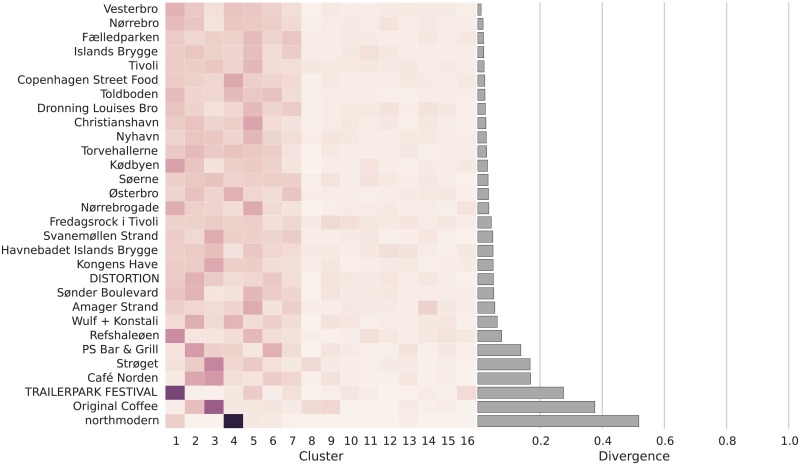
Visualization of place diversity in Copenhagen. The horizontal bar graph on the right shows the value of the divergence measure (DIV), with higher values indicating lower diversity. The heatmap in the middle indicates the relative presence or different clusters. Darker shaded cells shaded indicate greater presence of a cluster; white cells indicate complete absence.

Like in the case of Amsterdam, Copenhagen’s public parks and places are frequently sites of encounter (see Fig 5). Among the most cosmopolitan places are the Fælledparken (Commons Park) and the Tivoli, an amusement park. Users also frequently tag the neighborhoods of Vesterbro, Nørrebro, and the redeveloped harbor area Islands Brygge. These are not so much places as they are areas, so we cannot conclude that they are places of encounter. They are, however, areas that Instagram users across the board associate themselves with by tagging them in their posts.

The places with middling levels of diversity include hangouts popular among the younger users in clusters CPH2 and CPH3 (high school and college students), including a pedestrian shopping area and two bars in the downtown area. The most parochial places, finally, are the near-exclusive domain of a single cluster. The Trailerpark Festival, a music festival held in August, was tagged almost exclusively by users in cluster CPH1. Original Coffee, a coffee shop with locations in several neighborhoods, is the near-exclusive domain of CPH3 and two other clusters of teenage users, CPH8 and CPH9. Finally, Northmodern, a trade show dedicated to Danish design products, is overwhelmingly tagged by users in the Design & Family cluster (CPH4).

## Discussion

Urban researchers interested in social divisions traditionally have had to choose between two options that each have considerable tradeoffs. On the one hand, they could study social-spatial divisions quantitatively with the tradeoff that they had to ask questions that can be answered through data drawn from official records. On the other hand, they could study more complex and dynamic processes through which subcultures create and claim spaces, but then they had to resort to time and labor-intensive methods that can only be applied in a limited number of places or on limited samples. By using data drawn from social media, researchers of cities can begin to investigate at a very large scale and in minute detail how urban dwellers form groups within and through urban space. Instagram, in particular, offers extraordinary opportunities to users to showcase where they are and whom they associate with. Through its API and some of the methods we and others have developed, the medium also offers extraordinary opportunities for researchers interested in investigating segregation, the formation of subcultures, strategies of distinction, and status hierarchies.

Notwithstanding these opportunities, we should mention some caveats [58–61]. The lives of Instagram users are not contained within the platform, so our access to their lives is very much incomplete. The representations on Instagram, moreover, are highly selective. It would be mistaken to consider Instagram as somehow representative of the sum total of city dwellers’ uses of space. We should only look at Instagram if we are interested in what we can find there: the pictures and connections that *selectively* represent *selected* parts of the city from a *selective* group of urban dwellers. Our purpose was mainly to provide a “proof of concept” by developing a range of methods and demonstrating how Instagram data can shed new light on classic issues in the study of the city. While some of our findings would most likely prove robust (e.g., central public parks are widely popular and very cosmopolitan), other findings are more provisional. To benefit from the opportunities the data offer, it is necessary to carefully specify questions and complement Instagram with other sources of data.

## Supporting Information

S1 Fig Page Rank distributions of cluster subgraphs. (EPS)

## References

[1] Duggan M. Mobile messaging and social media 2015. Washington, D.C.: Pew Research Center; 2015 Available: http://www.pewinternet.org/2015/08/19/mobile-messaging-and-social-media-2015/

[2] McCracken H. Instagram’s all-new search & explore features will change how you use Instagram. Fast Company; 2015 Available: http://www.fastcompany.com/3047726/tech-forecast/with-new-search-explore-features-instagram-is-changing-how-youll-use-instagram

[3] Burke J, Estrin D, Hansen M, Parker A, Ramanathan N, Reddy S, et al. Participatory sensing. Proceedings of the world sensor web workshop. Boulder, Colo.: Association for Computing Machinery; 2006. pp. 1–5.

[4] Lane ND, Eisenman SB, Musolesi M, Miluzzo E, Campbell AT. Urban sensing systems: Opportunistic or participatory? Proceedings of the 9th workshop on mobile computing systems and applications. New York: Association for Computing Machinery; 2008. 10.1145/1411759.1411763

[5] Park RE. The city: Suggestions for the investigation of human behavior in the city environment. American Journal of Sociology. 1915;20: 577–612.

[6] Massey DS, Denton NA. American apartheid: Segregation and the making of the underclass. Cambridge, Mass.: Harvard University Press; 1998.

[7] O’Connor A, Tilly C, Bobo LD, editors. Urban inequality: Evidence from four cities. New York: Russell Sage Foundation; 2003.

[8] Charles CZ. The dynamics of racial residential segregation. Annual Review of Sociology. 2003;29: 167–207. 10.1146/annurev.soc.29.010202.100002

[9] Bellah RN, Madsen R, Sullivan WM, Swidler A, Tipton SM. Habits of the heart: Individualism and commitment in American life. Berkeley, Calif.: University of California Press; 1985.

[10] Maffesoli M. The time of the tribes: The decline of individualism in mass society. London: Sage; 1996.

[11] Hannerz U. Exploring the city. New York: Columbia University Press; 1980.

[12] Robson G, Butler T. Coming to terms with London: Middle class communities in a global city. International Journal of Urban and Regional Research. 2001;25: 70–86. 10.1111/1468-2427.00298

[13] Silver D, Clark TN, Yanez CJN. Scenes: Social context in an age of contingency. Social Forces. 2010;88: 2293–2324. 10.1353/sof.2010.0041

[14] Molotch H. Managed integration: Dilemmas of doing good in the city. Berkeley, Calif.: University of California Press; 1972.

[15] Blokland TV, van Eijk G. Do people who like diversity practice diversity in neighbourhood life? Neighbourhood use and the social networks of “diversity seekers” in a mixed neighbourhood in the Netherlands. Journal of Ethnic and Migration Studies. 2010;36: 313–332. 10.1080/13691830903387436

[16] Desmond M. Relational ethnography. Theory and Society. 2014;43: 547–579. 10.1007/s11186-014-9232-5

[17] Burawoy M. The extended case method: Four countries, four decades, four great transformations, and one theoretical tradition. Berkeley, Calif.: University of California Press; 2009.

[18] Batty M. The new science of cities. Cambridge, Mass.: MIT Press; 2003.

[19] Goodchild MF. Citizens as voluntary sensors: Spatial data infrastructure in the world of web 2.0. International Journal of Spatial Data Infrastructures. 2007;2: 24–32. Available: http://ijsdir.jrc.ec.europa.eu/index.php/ijsdir/article/view/28

[20] Bourdieu P. Photography: A middle-brow art. Palo Alto, Calif.: Stanford University Press; 1996.

[21] Lofland LH. The public realm: Exploring the city’s quintessential social territory. New York: Aldine De Gruyter; 1998.

[22] Sennett R. The fall of public man. Cambridge: Cambridge University Press; 1977.

[23] Anderson E. The cosmopolitan canopy: Race and civility in everyday life. New York: W.W. Norton; 2012.

[24] Talbot D. Regulating the night: Race, culture and exclusion in the making of the night-time economy. Aldershot: Ashgate; 2007.

[25] May RAB. Urban nightlife: Entertaining race, class, and culture in public space. New Brunswick, N.J.: Rutgers University Press; 2014.

[26] van Liempt I, van Aalst I, Schwanen T. Introduction: Geographies of the urban night. Urban Studies. 2015;52: 407–421. 10.1177/0042098014552933

[27] Hunter MA. The nightly round: Space, social capital, and urban black nightlife. City & Community. 2010;9: 165–186. 10.1111/j.1540-6040.2010.01320.x

[28] Hudson-Smith A, Crooks A, Gibin M, Milton R, Batty M. Neogeography and web 2.0: Concepts, tools and applications. Journal of Location Based Services. 2009;3: 118–145. 10.1080/17489720902950366

[29] Sui D, Goodchild M. The convergence of GIS and social media: Challenges for GIScience. International Journal of Geographical Information Science. 2011;25: 1737–1748. 10.1080/13658816.2011.604636

[30] Kelley MJ. The emergent urban imaginaries of geosocial media. GeoJournal. 2013;78: 181–203. 10.1007/s10708-011-9439-1

[31] Shelton T, Poorthuis A, Graham M, Zook M. Mapping the data shadows of hurricane sandy: Uncovering the sociospatial dimensions of “big data”. Geoforum. 2014;52: 167–179. 10.1016/j.geoforum.2014.01.006

[32] Zheng Y. Tutorial on location-based social networks. WWW 2012. Association for Computing Machinery; 2012. Available: http://research.microsoft.com/apps/pubs/default.aspx?id=163521

[33] Cranshaw J, Schwartz R, Hong JI, Sadeh N. The Livehoods project: Utilizing social media to understand the dynamics of a city. Sixth international AAAI conference on weblogs and social media. Dublin; 2012.

[34] Frias-Martinez V, Soto V, Hohwald H, Frias-Martinez E. Characterizing urban landscapes using geolocated tweets. Privacy, security, risk and trust. IEEE Computer Society; 2012. pp. 239–248. 10.1109/SocialCom-PASSAT.2012.19

[35] Silva TH, Vaz de Melo POS, Almeida JM, Salles J, Loureiro AAF. Visualizing the invisible image of cities. 2012 IEEE international conference on green computing and communications. 2012. pp. 382–389. 10.1109/GreenCom.2012.62

[36] Silva TH, Vaz de Melo POS, Almeida JM, Salles J, Loureiro AAF. Challenges and opportunities on the large scale study of city dynamics using participatory sensing. 2013 IEEE symposium on computers and communications. 2013. pp. 000528–000534. 10.1109/ISCC.2013.6755000

[37] Silva TH, Vaz de Melo POS, Almeida JM, Salles J, Loureiro AAF. A comparison of Foursquare and Instagram to the study of city dynamics and urban social behavior. Proceedings of the 2nd ACM SIGKDD international workshop on urban computing. New York: Association for Computing Machinery; 2013. pp. 1–8. 10.1145/2505821.2505836

[38] Silva TH, Vaz de Melo POS, Almeida JM, Salles J, Loureiro AAF. A picture of Instagram is worth more than a thousand words: Workload characterization and application. Proceedings of the IEEE international conference on distributed computing in sensor systems. Cambridge, Mass.: IEEE Computer Society; 2013. pp. 123–132. 10.1109/DCOSS.2013.59

[39] Hochman N, Schwartz R. Visualizing Instagram: Tracing cultural visual rhythms. Sixth international AAAI conference on weblogs and social media. Association for the Advancement of Artificial Intelligence; 2012.

[40] Schwartz R, Hochman N. The social media life of public spaces: Reading places through the lens of geo-tagged data Wilkena R, Goggin G, editors. Locative media. New York: Routledge; 2014;

[41] Hochman N, Manovich L. Zooming into an Instagram city: Reading the local through social media. First Monday. 2013;18 10.5210/fm.v18i7.4711

[42] Boy JD. Kijkeens: A tool for researchers. 2015. 10.5281/zenodo.34500

[43] Mitchell JC, editor. Social networks in urban situations. Manchester: Manchester University Press; 1969.

[44] Fischer CS. To dwell among friends: Personal networks in town and city. Chicago: Chicago University Press; 1982.

[45] Boyd D. It’s complicated: The social lives of networked teens. New Haven, Conn.: Yale University Press; 2014.

[46] Blondel VD, Guillaume J-L, Lambiotte R, Lefebvre E. Fast unfolding of communities in large networks. Journal of Statistical Mechanics: Theory and Experiment. 2008;2008: P10008.

[47] Csardi G, Nepusz T. The igraph software package for complex network research. InterJournal. 2006;Complex Systems: 1695. Available: http://igraph.org

[48] Traag VA. Louvain-igraph: Implementation of the Louvain algorithm for various methods for use with igraph in Python. 2015. 10.5281/zenodo.35117

[49] Salton G, Buckley C. Term-weighting approaches in automatic text retrieval. Information Processing & Management. 1988;24: 513–523. 10.1016/0306-4573(88)90021-0

[50] Uitermark J, Traag VA, Bruggeman J. Dissecting discursive contention: A relational analysis of the Dutch debate on minority integration, 1990–2006. Social Networks. Forthcoming 2016. Available: https://www.academia.edu/25777048

[51] Watts DJ, Strogatz SH. Collective dynamics of “small-world” networks. Nature. 1998;393: 440–442. 10.1038/30918 9623998

[52] Alstott J, Bullmore E, Plenz D. Powerlaw: A Python package for analysis of heavy-tailed distributions. PLoS ONE. 2014;9: 1–11. 10.1371/journal.pone.0085777 PMC3906378 24489671

[53] Brin S, Page L. The anatomy of a large-scale hypertextual web search engine. Computer Networks and ISDN Systems. 1998;30: 107–117.

[54] Duncan OD, Duncan B. A methodological analysis of segregation indices. American Sociological Review. 1955;20: 210–217.

[55] Massey DS, Denton NA. The dimensions of residential segregation. Social Forces. 1988;67: 281–315.

[56] Roberto E. Measuring inequality and segregation [Internet]. 2015. Available: http://arxiv.org/abs/1508.01167

[57] Abou-Moustafa KT. Divergence measures as diversity indices [Internet]. 2014. Available: http://arxiv.org/abs/1408.2863

[58] Boyd D, Crawford K. Critical questions for big data: Provocations for a cultural, technological, and scholarly phenomenon. Information, Communication & Society. 2012;15: 662–679. 10.1080/1369118x.2012.678878

[59] Manovich L. Trending: The promises and challenges of big social data In: Gold MK, editor. Debates in the digital humanities. Minneapolis, Minn.: University of Minnesota Press; 2012 pp. 460–475.

[60] Tufekci Z. Big questions for social media big data: Representativeness, validity, and other methodological pitfalls. Eighth international conference on weblogs and social media. Ann Arbor, Mich.: AAAI; 2014. Available: http://www.aaai.org/ocs/index.php/ICWSM/ICWSM14/paper/view/8062

[61] Kitchin R. The real-time city? Big data and smart urbanism. GeoJournal. 2014;79: 1–14. 10.1007/s10708-013-9516-8

